# High-Risk Areas for Congenital Zika Syndrome in Rio de Janeiro: Spatial Cluster Detection

**DOI:** 10.3390/tropicalmed9050105

**Published:** 2024-05-07

**Authors:** Danielle Amaral de Freitas, Mayumi Duarte Wakimoto, Sónia Dias, Reinaldo Souza-Santos

**Affiliations:** 1 Escola Nacional de Saúde Pública, Fundação Oswaldo Cruz, Rua Leopoldo Bulhões, Rio de Janeiro 1480, RJ, Brazil; dafufrj@hesfa.ufrj.br; 2 Instituto Nacional de Infectologia Evandro Chagas, Fundação Oswaldo Cruz, Av. Brasil, Rio de Janeiro 4365, RJ, Brazil; mayumi.wakimoto@ini.fiocruz.br; 3 National School of Public Health, Public Health Research Centre, Comprehensive Health Research Center (CHRC), REAL, NOVA University of Lisbon, Avenida Padre Cruz, 1600-560 Lisboa, Portugal; sonia.dias@ensp.unl.pt

**Keywords:** epidemiology, spatial analysis, cluster analysis, Zika virus infection, congenital Zika virus infection, congenital abnormalities, social vulnerability

## Abstract

Brazil reported 18,282 suspected congenital Zika syndrome (CZS) cases up to 2018 and accounts for 61.4% of the total reported Zika cases in the Americas in the period. To detect high-risk areas for children with CZS in the city of Rio de Janeiro, we used cluster detection and thematic maps. We analyzed data using a Poisson model in Satscan 10.1.3 software. We also analyzed the records of children with CZS from 2015 to 2016 to describe the clinical and epidemiological maternal and child profile, as well as live births in 2016 and the social development index (SDI) by neighborhood. In 2015 and 2016, the incidence rates of CZS were 8.84 and 46.96 per 100,000 live births in the city, respectively. Severe congenital findings such as microcephaly and brain damage, osteoarticular impairment, ocular abnormalities, and hearing loss were observed in 47 children. The spatial distribution of CZS was concentrated in the north and west zones in heterogeneous neighborhoods. The neighborhoods with the highest occurrence of CZS cases were found to have the worst SDIs. Stascan detected three spatial clusters in the north zone, where the SDI is lower. The clusters presented high relative risks for CZS (7.86, 1.46, and 2.08), although they were not statistically significant. Our findings highlight a higher occurrence of CZS in areas with less favorable socioeconomic conditions.

## 1. Introduction

Regional socioeconomic factors contributed to the higher likelihood of transmission of Zika virus between 2015 and 2016 in the Americas; comparing the countries, lower regional per capita gross domestic product (GDP) was associated with a higher risk of Zika virus spread [1].

The specific clinical diagnosis of Zika is difficult because it usually causes mild and self-limited disease or can develop as an asymptomatic infection in about 20% of cases [2,3]. However, Zika virus infection is associated with harmful outcomes for pregnancy and neonates. These outcomes include spontaneous abortion, fetal death, and several congenital malformations, mainly in the central nervous system, microcephaly, and osteoarticular and ocular changes characterizing congenital Zika syndrome (CZS) [4].

Congenital Zika syndrome has signs and symptoms similar to those found in other congenital infections described as STORCH, including toxoplasmosis, syphilis, chickenpox, parvovirus B1, rubella, cytomegalovirus, and herpes simplex [5,6,7,8]. 

In Brazil, it accounted for 61.4% of the total reported Zika cases in the Americas during the aforementioned period. Between 2015 and 2018, 18,282 suspected cases of developmental and growth-related changes associated with congenital Zika virus exposure were reported. The state of Rio de Janeiro was the fourth state with the highest number of confirmed cases (305) and reported 1214 suspected cases [9,10].

Forty-seven children were confirmed as CZS cases in the city of Rio de Janeiro between 2015 and 2016. The most frequent clinical signs were neurological manifestations, especially severe microcephaly. The children presented severe health conditions, which required specialized assistance, comprehensive care at different levels of healthcare, in addition to interinstitutional integration to meet all of the needs of the children and their families [11].

A few studies have shown the relationship between poverty and the high prevalence of CZS and microcephaly [12,13,14,15]. However, spatial distribution was only considered in the risk factor analysis in a single one [16].

This study aimed to detect spatial high-risk clusters of children with CZS, along with describing the clinical and epidemiological maternal and child profile in the city of Rio de Janeiro. In addition, we aimed to outline the spatial distribution of cases concerning the socioeconomic context between 2015 and 2016.

## 2. Materials and Methods

### 2.1. Design and Study Area

This was an ecological study based on spatial analysis, using the records of children notified as having CZS between 2015 and 2016 living in the city of Rio de Janeiro, Brazil. 

**Figure 1:**
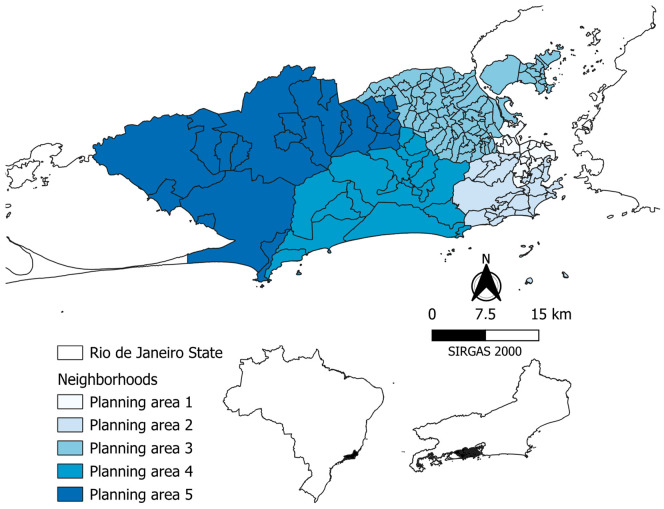
Map of the municipality of Rio de Janeiro according to neighborhoods and planning areas.

The city of Rio de Janeiro is the second-largest metropolis and the second-most populous city in Brazil, with high geographical and demographic diversity. The city is part of the major international tourism route in Brazil and Latin America. In 2022, it had 6,221,223 inhabitants and a high demographic density (5174.50 inhabitants/m^2^ in 2022), mainly in some specific areas characterized by slum complexes [17]. The municipality consists of 160 neighborhoods organized into five planning areas (PAs) (Figure 1):

PA 1: A total of 15 neighborhoods, including the downtown area and port region, representing 4.6% of the city’s population and 2.8% of the territory. Its net density is 7801 inhabitants per km^2^ (IBGE, 2012).

PA 2: A total of 25 neighborhoods, representing 17% of the population (997,478 inhabitants) and 8.2% of the territory. Its net density is 9932 inhabitants per km^2^. Luxury buildings (the South Zone of the city) and irregular occupation by low-income populations who have historically lived together.

PA 3: A total of 80 neighborhoods in the northern part of the city. it corresponds to 16.6% of the territory and 40.2% of the Rio de Janeiro population (2,353,590 inhabitants). Its net density is 11,567 inhabitants per km^2^. For every two slum dwellers in Rio de Janeiro, one is in PA 3.

PA 4: A total of 19 neighborhoods, which correspond to 24% of the territory and 11.6% of the population (682,051 inhabitants) of Rio de Janeiro. Its net density is 2322 inhabitants per km^2^. It is located in the west of the city and had high population growth (29.6%) between 1991 and 2000, with a heterogeneous HDI (human development index).

PA 5: A total of 20 neighborhoods in the western part of the city. It corresponds to 48.4% of the municipality’s territory and 26.6% of the population of Rio de Janeiro (1,556,505 inhabitants). Its net density is 2627 inhabitants per km^2^, where there were formally extensive properties for agricultural purposes, which ceased to exist with the pressure of urbanization.

### 2.2. Data Source and Analysis

The analysis considered records of children with CZS notified between 2015 and 2016. As recommended by the Brazilian Ministry of Health, the notifications took into account laboratory confirmation or clinical–epidemiological criteria (imaging examinations of the child, clinical history of the mother and child, and epidemiological history) [18]. All data, including the addresses of the children, were obtained from the Public Health Event Registration System (PHER). There were limited social and demographic data on the cases in the information system.

### 2.3. Congenital Zika Syndrome Notification

Notifications of suspected cases of association between microcephaly and congenital exposure to Zika virus began in week 45 of 2015, under the surveillance and response protocol for the occurrence of microcephaly and/or changes in the central nervous system. From then on, states and municipalities adapted the health surveillance network and the healthcare network for the notification and monitoring of cases. Data were collected from children, who were residents of the city of Rio de Janeiro, notified in the PHER, with a confirmed diagnosis of CZS according to laboratory criteria (RT-PCR of the mother or child’s serum or urine) and clinical–epidemiological criteria (imaging exams of the child and clinical and epidemiological history of the mother and child) according to the classification of integrated surveillance and healthcare guidelines within the scope of the Public Health Emergency of National Importance [18,19].

### 2.4. Maternal and Child Profile Associated with Congenital Zika Syndrome

To characterize the maternal and child profile associated with CZS, all maternal variables were extracted from the PHER: date of birth, race/color, date of onset of symptoms, signs and symptoms, period of gestation of Zika virus infection (trimester), and neighborhood of residence; variables relating to children: sex, full-term pregnancy, anthropometric measurements of the child (weight, length, and head circumference—HC), classification of the fetus (term or pre-term), period of microcephaly detection, history of microcephaly diagnosis, and other congenital changes; confirmation criteria; and clinical evolution. The mother’s age was stratified into age groups of 10 years, the children’s weight was classified as greater or less than 2500 g at birth, the length as greater or less than 48 cm at birth, and the HC greater or lesser than 31.5 cm at birth. The conformity of the anthropometric measurements of the children at birth in relation to the gestational age at birth, in weeks, was verified based on strata of the standard deviation of the Z-score using the Intergrowth-21 criterion [20,21]. A descriptive analysis of the data was performed using SPSS 20.0 software. 

### 2.5. High-Risk Clusters of Children with CZS: Spatial Distribution in the Socioeconomic Context

The addresses of the children were georeferenced in two ways: (1) by the centroids of the neighborhoods of households to detect clusters and (2) by the geographic coordinates of the households, collected with Google Maps, for point pattern analysis. To detect spatial clusters and relative risks (RRs), we used the Kuldorff scan statistic, with a discrete Poisson model in Satscan 10.1.3 software. The circular window of the cluster was limited to 50% of the population at risk and a radius of 8 km in the search for high-rate clusters [22,23]. Cases of CZS and the total number of live births were georeferenced by the centroids of the neighborhoods. The number of live births in each neighborhood of Rio de Janeiro in 2016 was obtained from the Pereira Passos Institute information system for Rio de Janeiro city [24].

The probability function was maximized in all locations and window sizes, and the function with the maximum probability constitutes the most likely cluster, that is, the cluster least likely to be due to chance. The likelihood ratio for this window is the statistic of the maximum likelihood ratio test. The *p*-value was obtained through Monte Carlo simulation, comparing the classification of the maximum probability of the real data set with the maximum probabilities of the random data sets [22,23].

To calculate the RR, the radius of the cluster that had the highest risk for the event studied was compared with its neighbors in the study area. The RR refers to the incidence of children with CZS within the cluster compared with the incidence outside the cluster [22,23,25].

To find out the differences in socioeconomic conditions among the different neighborhoods of the city, a thematic map was elaborated with the social development index (SDI). The SDI is a composite indicator that represents four main dimensions: housing conditions, sanitation, education, and income, based on eight indicators from the 2010 demographic census. The SDI ranges from 0 to 1, with 0 accounting for places with the worst socioeconomic conditions and 1 accounting for places with the best ones [26].

The indicators used to calculate the SDI are: the percentage of permanent private households with adequate water supply, that is, connected to the general distribution network; the percentage of permanent private households with adequate sewage, that is, connected to the general sewage or pluvial network; the percentage of permanent private households with garbage collected directly by cleaning services or placed in cleaning service buckets; the average number of bathrooms per resident (numerator = number of bathrooms in the permanent private household; denominator = total number of people in the permanent private household); the percentage of illiteracy among residents aged 10 to 14 in relation to all residents aged 10 to 14 years; the per capita income of permanent private households, expressed in 2010 minimum wages; the percentage of private households with per capita household income up to one minimum wage; and the percentage of private households, with the per capita household income exceeding five minimum wages [26].

The results by neighborhoods were contextualized by PAs and areas of the city. This approach allows for a better comparison with SDI distribution. The layers were superimposed to identify possible visual associations between the clusters and the SDI.

This study was approved by the Ethics Committees of the National School of Public Health, Oswaldo Cruz Foundation (CAAE: 94162218.5.0000.5240), and the Municipal Health Secretariat of Rio de Janeiro (CAAE: 94162218.5.3001.5279).

## 3. Results

Between 2015 and 2016, 14,764 pregnant women with suspected Zika virus infection were reported in Rio de Janeiro and 4380 (30%) cases were confirmed. The most frequent neighborhoods of confirmed cases were PA 3 (39%) and 5 (35%), which are the most populous and poor areas of the city.

Among the reported pregnant women, 8% (n = 47) had children with congenital Zika syndrome, with most of them aged between 21 and 30 years (60%), non-White (58%), and with Zika diagnosis confirmed in the first trimester of pregnancy (75%) (Table 1).

In 2015, the incidence of CZS was 8.84 per 100,000 live births, and in 2016, it was 46.96 per 100,000 live births. No child with CZS was born in 2017.

Children had on average low weight and height, with a head circumference < 31.5 cm (81%), and most of them exhibited severe microcephaly with Z-scores < −3 standard deviations (49%). Among the detected congenital abnormalities, brain alterations (89%), microcephaly (85%), and osteoarticular and ocular abnormalities (19.1%) were the most frequent ones. Most of the children had microcephaly (87%), 42% had brain and other systemic alterations, 45% had brain abnormalities, and 13% did not have microcephaly but had other congenital abnormalities. Six children died, (four) most in the post-neonatal period (Table 1).

**Figure 2:**
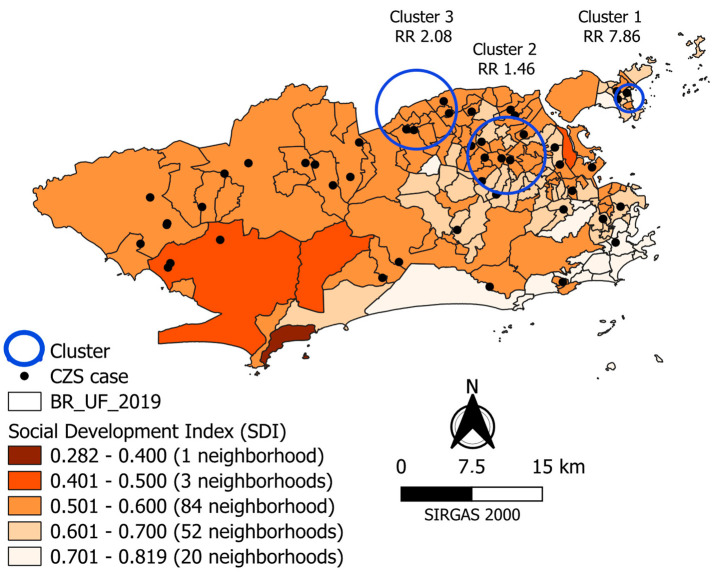
Distribution of the social development index, cases of congenital Zika syndrome (2015–2016), and spatial clusters according to the neighborhoods of the city of Rio de Janeiro.

The spatial distribution of CZS cases was heterogeneous in Rio de Janeiro, with an accumulation in the neighborhoods of PAs 3 (20) and 5 (15) (Figure 2 and Table 2). The spatial distribution of the SDI was also heterogeneous. The highest SDI variation by neighborhood was in PA 4, where we find the Grumari neighborhood, and the lowest SDI was in the municipality (0.282). The best SDIs (above 0.701) were in the neighborhoods of PAs 2, 3, and 4, in the south, north, and west zones of the city, respectively.

The visual analysis showed that the neighborhoods with the highest distribution of confirmed cases of children with CZS are the same districts with the worst SDI. Three children with CZS were in neighborhoods with a worse SDI (range 0.401 to 0.500) and none were in the districts with a better SDI (Figure 2).

The analysis detected three spatial clusters, all located in PA 3, in the neighborhoods of the northern zone of Rio de Janeiro, where the SDI ranges from 0.50 to 0.70, and the average was 0.60 (Table 2 and Figure 2).

Cluster 1 is located on Ilha do Governador (PA 3), with a radius of 1.47 km, 695 live births, and three cases of CZS. It has a relative risk of 7.86 (*p*-value = 0.71). Cluster 2 is in the central region of PA 3. It has a radius of 4.11 km, 8630 live births, and seven cases of CZS. The relative risk is 1.46, and the *p*-value is 0.71. Cluster 3 is also in PA 3 and overlaps some neighborhoods in PA 5. It has a radius of 4.25 km, 3463 live births, and four cases of CZS. The relative risk is 2.08 (*p*-value = 0.84) (Table 3 and Figure 2).

## 4. Discussion

The distribution of CZS cases is similar to the spatial pattern of the worst SDI in Rio de Janeiro. The cases mainly occur in the neighborhoods of the northern and western areas of the city. A study conducted between 2008 and 2014, before the introduction of the chikungunya and Zika viruses, found that the western and northern zones of the city were also high-risk areas for dengue in children under five years old [27]. This indicates that these regions have a history of high incidence rates of arboviruses. Areas of the city with a high number of confirmed cases in pregnant women also had a high distribution of CZS cases. Additionally, the areas with the highest risk of CZS cases are the poorest in the city.

### 4.1. Maternal and Child Profile Associated with Congenital Zika Syndrome

Congenital Zika syndrome is a severe condition with a 13% fatality rate among the children in this study. It also leads to congenital alterations that impact the motor and cognitive development of children, causing them to be dependent even for basic life needs [28,29]. Most mothers of children who did not survive had signs and symptoms of Zika in the first trimester of pregnancy, a fact highlighted in studies that associate the severity of the syndrome with maternal infection in the first trimester of pregnancy [30,31,32]. 

Our results showed a small incidence of CZS in the city of Rio de Janeiro. In comparison to syphilis, the number of cases of CZS was 189 times lower in 2015 and 87 times lower in 2016 [33]. However, factors such as reduced access to diagnosis, either RT-PCR or imaging exams, for suspected children should be considered [34]. Additionally, the interval between notification of the case and the onset of signs and symptoms impairs the collection of samples in a timely manner and may result in the underreporting of cases [11,35]. 

Although most children were born at full term, prematurity occurred in 28% of the cases, which is 2.25 times higher than that observed in the city of Rio de Janeiro in 2016 (12.3%) [36]. The weight and length of the children were within the normal range according to the Intergrowth scale for gestational age and sex. However, the average weight and length of the children were low, indicating that they tend to have lower height and weight [20,21].

Microcephaly was present in 85%, mostly (n = 23) severe microcephaly, at more than three standard deviations below the ideal according to sex and gestational age, based on the Intergrowth scale [20,21]. A small number of children presented brain damage such as ventriculomegaly and calcifications as observed in other studies [8,30,31,37,38,39,40,41,42,43,44,45,46,47,48,49,50,51,52,53] and also noted in a systematic review [4]. 

Ocular abnormalities were found in 9% of the children. Other studies have also identified visual, anatomical, and functional alterations in children with CZS [30,32,39,52,54,55,56,57,58,59,60,61].

Osteoarticular alterations (9%) were frequent, as observed in other studies. The musculoskeletal system was the second most affected system in newborns exposed to Zika virus in utero, with them presenting arthrogryposis [30,49,62,63,64,65]. 

We observed a small number of congenital abnormalities in systems other than the nervous system, as identified in other studies [5,8,30,31,32].

Children with CZS exhibit severe delays in neurological development and slower growth rates, indicating delays in all Bayley domains, and even with the incorporation of a unique care plan, they show limited improvement in development with motor impairment consistent with cerebral palsy [66,67,68]. The impacts of the disease on children and their families are diverse and encompass the following dimensions: (a) social changes in family life plans; (b) subjective experiences leading to overload and uncertainties; (c) economic and material impacts with loss of income and increased expenses; and (d) impacts on the health of both children and families, as well as access to healthcare services [69].

Children with CZS are, therefore, critically ill children who require comprehensive care at different levels of healthcare, ranging from basic to specialized assistance, in addition to interinstitutional integration to meet all of the needs of the children and their families [11].

In Brazil, the healthcare system is universal and free of charge. However, there are reports of barriers in accessing medical supplies such as anticonvulsants, diapers, and nutritional formulas, resulting in increased costs for families [70]. Additionally, studies point out weaknesses in prenatal care follow-up due to insufficient coverage and limited access to diagnostic support networks. The same applies to children with CZS due to the inability of primary healthcare to implement the essential attributes for comprehensive care and the lack of coverage of specialized health services necessary for children with CZS and cerebral palsy [11,71,72].

The number of non-White women who had children with the syndrome was higher compared to White women, which is consistent with other studies that have observed the high prevalence of microcephaly associated with or without Zika infection during pregnancy. This could be attributed to the socioeconomic vulnerabilities to which these women are exposed [14,59,67,73,74]. Another important proxy for socioeconomic conditions is education, which was not available in our study. However, other studies associate lower educational levels with a higher risk of CZS [67,74].

### 4.2. High-Risk Clusters of Children with CZS: Spatial Distribution in the Socioeconomic Context

Rio de Janeiro has extreme heterogeneity among its districts, with unequal distribution of urban structures, housing, and income. Other studies have found that in vulnerable areas, there is also an increased burden of disease, especially communicable diseases such as dengue, chikungunya, and Zika, maintaining high rates of building infestation of the vector [75,76,77,78].

The results of a study carried out in Recife corroborate the association of social vulnerability with arboviruses. A higher risk of microcephaly was found in areas with the lowest municipal human development index, a poor sewage system, and irregular garbage collection rates, in addition to the highest density of vector mosquito larvae [14]. Usually, these areas have a higher population density and place families in vulnerable situations, without support to meet all the needs of a syndromic child [79,80,81,82]. Studies conducted in other Brazilian cities corroborate these results [70,83].

In our study, Grumari is the neighborhood with the lowest SDI. It is worth mentioning that this neighborhood has a low demographic density; it is a small area with few residences and services and is less urbanized. According to Cavallieri [84], the SDI is a composite indicator and can be influenced by population size and infrastructure services. Therefore, it is necessary to consider some scenarios, such as the neighborhood of Grumari.

Regarding the three spatial clusters of high risk detected, they are located in the neighborhoods of PA 3, north of Rio de Janeiro. This region has a large number of urban areas, a high population density, poor urban structure, and intense urban violence. There are records of a high number of homicides, assaults, and deaths due to police intervention in this area [85]. A similar scenario was also observed in northeastern Brazil [86]. Studies have found a higher prevalence of microcephaly among residents of areas with poor living conditions compared to those with better conditions and a strong correlation with the poverty rate [12,14].

The RR interpretation must be performed with caution in our study. The most likely cluster (cluster 1) had the highest RR (7.86), the smallest area, and the lowest live births. The two other clusters were larger, with higher live births than cluster 1. The RRs were 1.46 and 2.08 in clusters 2 and 3, respectively. However, none of the three clusters were significant. This result may be related to the low incidence of the CZS concerning the number of live births. However, these results are relevant because they show areas with a high risk of CZS. Similar results have also been observed [78].

Space–time analysis carried out between 2015 and 2016 detected clusters of dengue, Zika, and chikungunya simultaneously in Rio de Janeiro. These clusters were in neighborhoods with a high population density and low socioeconomic status, generally located in the north and west. Although the Zika clusters had higher relative risks in the west of the city, the first cluster was detected in the north [78].

There is a possible temporal association between clusters in the northern zone and the period of exposure of pregnant women, since affected children in this region were born up until September 2016. Another study pointed out the simultaneous circulation of arboviruses in pregnant women in Rio de Janeiro [76]. This result corroborates the temporality of the infection period hypothesis.

Similar results were found in a study in Australia about dengue. The authors identified high relative risks in regions with greater social vulnerability and greater vector exposure, with the same mosquito vector as Zika [87]. A study that investigated the relationship between Zika virus and environmental factors indicated that social factors had a greater influence than natural factors on the spread of it. The authors showed that population density has the greatest impact and is the key factor in the spread of Zika virus in South America [88]. Souza et al. [14] found that newborns with microcephaly who lived in the poorest areas of the city of Recife had a high relative risk of microcephaly development.

Other studies that used the spatial cluster detection methodology analyzed the relative risks to climatic characteristics and mosquito vectors [87,89]. This type of analysis is relevant for the real-time monitoring of disease dispersion, especially arboviruses for the allocation of financial resources and services in areas with the highest risk. Despite this, the method is not widely used to detect the risk of diseases such as CZS. Therefore, we highlight the importance of studies to identify the spatial risk and know the territorial structure and health and social demands of children and their families.

The main limitation of our study was the use of secondary data. The system (PHER), created in light of the Zika epidemic, has limited fields and gaps regarding clinical, laboratory, and imaging information. Another limitation is that it was not possible to verify sexual transmission among pregnant women with confirmed Zika virus infection because there was not enough evidence of this transmission route during the years of study. However, a slight reduction in the number of live births was observed in the subsequent years in Rio de Janeiro, which may be related to the fear of infection during pregnancy along with the actions taken by the healthcare system regarding the use of repellents, condoms, and family planning. However, to our knowledge, no similar studies have been published targeting this specific population.

## 5. Conclusions

The incidence of arboviruses is influenced by factors related to (1) urban structure with adequate housing conditions, including sanitation, running water, and regular garbage collection; (2) individual and collective conditions that can increase the number of mosquito vector reservoirs, such as the level of education and behavior of the population with practices that can increase vector reservoirs; and (3) environmental factors and climate change. Rio de Janeiro is a city with a vast and heterogeneous territory and high population density that is prone to important climate changes with heat waves that can modify the proliferation profile of the mosquito vector, in addition to increasing the burden of disease for the population in the near future [79]. Therefore, it is necessary to map and monitor risks and establish plans to reduce the burden of disease, especially in cases with such disastrous consequences for children, families, communities, and health services, such as congenital Zika syndrome.

The profile of children with SCZ living in the city of Rio de Janeiro is similar to that found in other studies. They portray children with congenital anomalies capable of causing a high level of dependence, a condition that demands comprehensive and specialized attention with broad assistance and social support [5,7,8,90,91]. However, access to healthcare for children with CZS is difficult due to the social vulnerability observed in these families and barriers to accessing the healthcare network; insufficient and fragmented assistance; a lack of communication between the various specialized services and the different levels of care; and a lack of a health system referral and counter-referral network [11,70,74,79,80,82,92,93]. Furthermore, it is difficult to access health units due to distance, generating greater costs and time expenditure, especially in places with greater social vulnerability [11,70,74,79,80,82,92].

Monitoring the occurrence and severity profiles of cases of arboviruses and congenital Zika syndrome, as well as using spatial analysis in the routine work process of health surveillance, is an essential point in increasing the sensitivity of capturing cases in a timely manner and promoting an early response to possible public health emergencies. Therefore, strategies that integrate interinstitutional actions are needed, both to reduce vulnerability in poorer regions and to promote access to urban social and health services in order to reduce the burden of disease and improve quality of life.

## Figures and Tables

**Table 1:** Clinical and epidemiological profile of mothers and children with congenital Zika syndrome (CZS), 2015 to 2016, in Rio de Janeiro.

**Mother Characteristics**					
Neighborhood Residence (PA)	n	%	Pregnancy in Which Zika Infection Occurred (Trimester)	n	%
1	5	11	First	35	75
2	3	6	Second	6	13
3	20	43	Third	1	2
4	4	9	Ignored	5	11
5	15	32			
Total	47	100			
Age (years)	n	%	Race/color	n	%
≤20	6	13	Non-White	27	58
21 to 30	22	47	White	19	40
31 to 40	16	34	Ignored	1	2
>40	3	6			
**Children with CZS characteristics**					
Gestational age at birth	n	%	Weight	n	%
Term	34	72	<2500 g	20	43
Premature	13	28	2500 g or more	27	57
Height	n	%	Cephalic perimeter	n	%
<48 cm	34	72	<31.5 cm	38	81
48 cm or more	13	28	>31.5	7	15
			Ignored	2	4
Z-score	n	%	Evolution	n	%
<−3	23	49	In follow-up	41	87
−2 to −3	7	15	Death	6	13
−1.9 to 1.9	7	15	Early neonatal	1	
2 to 3	1	2	Late neonatal	1	
Ignored	9	19	Post-neonatal	4	
Congenital abnormalities				n	%
Brain				42	89
Microcephaly				40	85
Osteoarticular				9	19
Ocular				9	19
Auditory				7	15
Development				7	15
Hydrocephalus				6	13
Kidneys				3	6
Uterine growth restriction				3	6
Hepatosplenomegaly				3	6
Lungs				2	4
Cardiac				1	2
Liver				1	2
Final diagnosis				n	%
Microcephaly, brain changes, and changes in other systems				20	43
Microcephaly and brain changes				21	45
No microcephaly, with other congenital changes.				6	13

**Table 2:** Children with congenital Zika syndrome (2015–2016) and social development index in the city of Rio de Janeiro, according to the planning area of this city.

PA	CZS	Social Development Index					
		Districts	Average	SD	Median	Minimum	Maximum
1	5	15	0.59	0.03	0.60	0.54	0.64
2	3	25	0.71	0.07	0.72	0.53	0.82
3	20	80	0.60	0.04	0.59	0.50	0.72
4	4	19	0.60	0.11	0.59	0.28	0.77
5	15	21	0.56	0.05	0.56	0.49	0.70
Total	47	160	0.61	0.07	0.59	0.28	0.82

PA—planning area; CZS—congenital Zika syndrome.

**Table 3:** Spatial clusters of children with congenital Zika syndrome who were residents of the municipality of Rio de Janeiro between 2015 and 2016.

	Cluster 1	Cluster 2	Cluster 3
Radius	1.47 km	4.11 km	4.25 km
Live births (population)	695	8630	3463
CZS	3	7	4
Relative risk	7.86	1.46	2.08
Likelihood ratio	3,490,937	3,482,130	2,894,695
*p*-value	0.71	0.71	0.84

## Data Availability

Data are available upon request only due to ethical restrictions.
